# The Medical Impact of Hepatitis D Virus Infection in Natives and Immigrants: The Italian Paradigm

**DOI:** 10.1111/liv.70242

**Published:** 2025-07-29

**Authors:** Gian Paolo Caviglia, Eleonora Dileo, Antonella Olivero, Giulio Mengozzi, Alessia Ciancio, Alessandro Loglio, Mauro Viganò, Stefano Fagiuoli, Piero Colombatto, Barbara Coco, Maurizia Rossana Brunetto, Debora Angrisani, Alfonso Galeota Lanza, Carlo Magni, Giuliano Rizzardini, Vincenzo Messina, Michele Milella, Annalisa Saracino, Letizia Marinaro, Giuseppe Cariti, Valentina Cossiga, Filomena Morisco, Silvia Cretella, Gabriella Verucchi, Elisa Biliotti, Giampiero D'Offizi, Marcello Feasi, Emanuele Pontali, Carmine Coppola, Rachele Rapetti, Mario Pirisi, Antonio Izzi, Teresa Santantonio, Vito Di Marco, Aldo Marrone, Nicola Coppola, Marco Rizzi, Anna Maria Cattelan, Grazia Anna Niro, Pierluigi Toniutto, Francesca Romana Ponziani, Luca Saverio Belli, Alessandro Federico, Gaetano Bertino, Michele Barone, Massimo Puoti, Alberto Civolani, Marco Distefano, Lorenzo Antonio Surace, Raffaele Cozzolongo, Ester Marina Cela, Elisabetta Teti, Pietro Lampertico, Tommaso Stroffolini, Mario Rizzetto, the HDV Describe Study Group, Alessandra Risso, Yulia Troshina, Giorgio Maria Saracco, Tilde Manetta, Barbara Donati Marello, Paola Merlach, Elisa Farina, Giovan Giuseppe Di Costanzo, Raffaella Tortora, Spinello Antinori, Matilde Quaranta, Silvia Taurian, Caterina Quarta, Annarosa Garbuglia, Nicolina Capoluongo, Alessandro Perrella, Nunzia Cuomo, Francesco Tortorici, Emanuele Bracciamà, Clelia Cosentino, Mariantonietta Pisaturo, Giuliana Cologni, Francesco Barbaro, Rosa Cotugno, Ezio Fornasiere, Maria Pallozzi, Raffaella Viganò, Marcello Dallio, Mario Romeo, Laura Rapisarda, Enrico Siciliano, Alfredo Risicato, Elisabetta Falbo, Massimo Andreoni, Maria Paola Anolli

**Affiliations:** ^1^ Department of Medical Sciences University of Torino Turin Italy; ^2^ Division of Gastroenterology Hepatology and Transplantation, ASST Papa Giovanni XXIII Bergamo Italy; ^3^ Department of Medicine, Gastroenterology University of Milan Bicocca Milan Italy; ^4^ Department of Clinical and Experimental Medicine, Hepatology and Liver Physiopathology Laboratory and Internal Medicine Unit University of Pisa Pisa Italy; ^5^ Hepatology Unit, AORN A. Cardarelli Naples Italy; ^6^ Division of Infectious Diseases ASST‐FBF‐Sacco Milan Italy; ^7^ Infectious Diseases Unit, AORN Sant'Anna e San Sebastiano Caserta Italy; ^8^ Clinic of Infectious Diseases, Department of Precision and Regenerative Medicine and Ionian Area, University of Bari “Aldo Moro” Bari Italy; ^9^ Clinica Malattie Infettive UniTo, “Ospedale Amedeo di Savoia” Turin Italy; ^10^ Department of Clinical Medicine and Surgery Liver and Biliary Diseases Unit, University of Naples “Federico II” Naples Italy; ^11^ Infectious Diseases Unit, Department for Integrated Infectious Risk Management IRCCS Azienda Ospedaliero‐Universitaria di Bologna Bologna Italy; ^12^ Infectious Diseases Unit, Department of Medical and Surgical Sciences University of Bologna Bologna Italy; ^13^ Infectious Diseases and Hepatology Unit, National Institute for Infectious Diseases Lazzaro Spallanzani IRCCS Rome Italy; ^14^ Department of Infectious Diseases Galliera Hospital Genoa Italy; ^15^ Unit of Hepatology and Interventional Ultrasonography, Department of Internal Medicine OORR Area Stabiese Gragnano Italy; ^16^ Department of Translational Medicine (DiMeT), Internal Medicine, Azienda Ospedaliero‐Universitaria “Maggiore Della Carità” Università del Piemonte Orientale Novara Italy; ^17^ UOC di Malattie Infettive Emergenti e Ad Alta Contagiosità, Ospedale Cotugno Naples Italy; ^18^ Infectious Diseases Unit, Department of Medical and Surgical Sciences University of Foggia Foggia Italy; ^19^ Section of Gastroenterology and Hepatology, Dipartimento Di Promozione Della Salute, Materno Infantile, Medicina Interna e Specialistica Di Eccellenza (PROMISE) University of Palermo Palermo Italy; ^20^ Department of Advanced Medical and Surgical Sciences University of Campania “Luigi Vanvitelli” Naples Italy; ^21^ Infectious Diseases Unit, Department of Mental Health and Public Medicine University of Campania “Luigi Vanvitelli” Naples Italy; ^22^ SC Infectious Diseases, ASST Papa Giovanni XXIII Bergamo Italy; ^23^ Infectious and Tropical Diseases Unit, Padua University Hospital Padua Italy; ^24^ Gastroenterology Unit, Fondazione Casa Sollievo Della Sofferenza IRCCS San Giovanni Rotondo Italy; ^25^ Hepatology and Liver Transplant Unit, Azienda Sanitaria Universitaria Integrata di Udine Udine Italy; ^26^ Liver Unit, Centro Malattie Dell'apparato Digerente (CEMAD), Medicina Interna e Gastroenterologia, Fondazione Policlinico Universitario Gemelli IRCCS Rome Italy; ^27^ Hepatology and Gastroenterology Unit, ASST Grande Ospedale Metropolitano Niguarda Milan Italy; ^28^ Department of Precision Medicine University of Campania “Luigi Vanvitelli” Naples Italy; ^29^ Department of Clinical and Experimental Medicine, Hepatology Unit, University Hospital Policlinico “G. Rodolico ‐ San Marco” University of Catania Catania Italy; ^30^ Gastroenterology Unit, Department of Precision and Regenerative Medicine—Jonian Area—(DiMePRe‐J), University of Bari “Aldo Moro” Policlinic University Hospital Bari Italy; ^31^ Division of Infectious Diseases, Department of Medicine, ASST Grande Ospedale Metropilitano Niguarda University of Milan Bicocca Milan Italy; ^32^ Teaching Hospital “Duilio Casula”, Azienda Ospedaliero‐Universitaria di Cagliari Cagliari Italy; ^33^ UOSD Hepatology, Ospedale Umberto I°, ASP 8 Siracusa Siracusa Italy; ^34^ Dipartimento Di Prevenzione, Azienda Sanitaria Provinciale di Catanzaro, Centro di Medicina del Viaggiatore e Delle Migrazioni Lamezia Terme Italy; ^35^ Gastroenterology Unit, IRCCS “S. De Bellis” Castellana Grotte Italy; ^36^ Gastroenterology Unit, Riuniti Polyclinic of Foggia Foggia Italy; ^37^ Infectious Disease Unit, Department of Systems Medicine University of Rome Tor Vergata Rome Italy; ^38^ Division of Gastroenterology and Hepatology Foundation IRCCS Ca' Granda Ospedale Maggiore Policlinico Milan Italy; ^39^ Department of Pathophysiology and Transplantation, CRC “A. M. and A. Migliavacca” Center for Liver Disease University of Milan Milan Italy; ^40^ Department of Tropical and Infectious Diseases Policlinico Umberto I Rome Italy

**Keywords:** chronic hepatitis D, cirrhosis, epidemiology, HDV, HDV‐RNA

## Abstract

**Background and Aim:**

Ongoing migratory flows are reconstituting the hepatitis D virus (HDV) reservoir in Italy. We aimed to characterise the current clinical and virologic features of HDV infection in both native Italians and migrants.

**Methods:**

We enrolled 515 hepatitis B surface antigen (HBsAg)‐positive patients with detectable anti‐HDV antibodies from 32 Italian centres between August 2022 and July 2024; all patients underwent centralised virologic assessment.

**Results:**

Overall, 432 out of 515 (83.9%) patients were HDV‐RNA‐positive (4.39, 1.30–5.82 Log IU/mL; 99.0% HDV genotype‐1). HDV‐RNA levels correlated with ALT (*r*_s_ = 0575, 0.514–0.630) and hepatitis B core‐related antigen (*r*_s_ = 0.521, 0.455–0.581). Native Italians (*n* = 317; 61.6%) were older than migrants (*n* = 198; 38.4%) (median age: 60, 55–65 vs. 46, 39–54 years; *p* < 0.001) and were more frequently male (68.1% vs. 49.5%; *p* < 0.001), with a higher prevalence of liver cirrhosis (70.3% vs. 50.5%; *p* < 0.001) and hepatocellular carcinoma (14.8% vs. 0.5%; *p* < 0.001). Among Italians, 223 (70.3%) had liver cirrhosis, 46 (14.5%) had chronic hepatitis D (CHD) without cirrhosis and 48 (15.1%) exhibited inactive/minimal disease with low viremia (≤ 3 Log IU/mL). Among migrants, 100 (50.5%) had liver cirrhosis, 58 (29.3%) had CHD and 40 (20.2%) showed inactive/minimal disease with low viremia (≤ 3 Log IU/mL).

**Conclusions:**

The current clinical landscape of chronic HDV infections in Italy is heterogeneous, changing the perspective of CHD as uniformly severe; although cirrhosis remains common, a substantial proportion of both native Italians and migrants present with milder forms of disease.

**Summary**

- Chronic HDV infection in Italy increasingly affects younger, non‐native individuals, mainly Eastern European immigrants, with milder liver disease; prevalence is higher in Central‐Northern regions.
- Most patients (83.9%) are HDV‐RNA positive, predominantly genotype‐1.
- A subgroup of non‐cirrhotic patients presented with inactive/minimal disease, exhibiting normal ALT, lower HDV‐RNA, HBsAg, HBcrAg and liver stiffness, but no differences in demographics, cofactors or prior IFN exposure compared to active chronic hepatitis D cases.

## 1 Introduction

In the last 30 years, the introduction of the hepatitis B virus (HBV) vaccine has led to optimal control of HBV infection in the industrialised world, resulting in a great decline in new hepatitis D virus (HDV) infections. However, the prospect of eradicating hepatitis D is hindered by the influx of HDV‐infected migrants, who are creating a new virus reservoir in Europe [1]. Italy provides a valuable setting to observe these changes, as hepatitis D was endemic when HDV was first described in 1977 [2], and the characteristics of Italians with this infection have been updated at 10‐year intervals to the present [3, 4, 5]. The introduction of the HBV vaccine in 1991 for newborns and adolescents (for the following 12 years) has provided immunity to all Italians aged 45 and under, virtually eliminating HBV‐dependent hepatitis D in the younger domestic generations who were previously at the highest risk. As a result, the clinical profile of chronic hepatitis D (CHD) patients has shifted from a predominance of rapidly progressive liver disease leading to hepatic failure to a more heterogeneous spectrum, often including older patients with compensated cirrhosis or indolent disease. This likely reflects a selection bias related to improved survival and earlier diagnosis [5]. Meanwhile, HDV infections among immigrants are on the rise [6], posing the challenge of potentially severe hepatitis D in marginalised populations.

Although the natural history of HDV in native Italians was previously documented, the medical impact of HDV in immigrants remains largely unknown. To define and compare the contemporary medical characteristics of HDV infection, we established a national collaborative study aimed at determining the clinical/virologic profiles of HBsAg carriers with anti‐HDV antibodies, recruited across Italy between 2022 and 2024, including both native Italians and immigrants born abroad.

## 2 Materials and Methods

### 2.1 Study Design

In this cross‐sectional study, we included consecutive adult patients (> 18 years) positive for HBsAg and anti‐HDV enrolled across 32 Italian liver centres between August 2022 and July 2024 (ClinicalTrials.gov: NCT05723068). Eligible were both patients first diagnosed during the study period and those already on follow‐up.

The study protocol was compliant with the principles of the 1975 Declaration of Helsinki; it was approved by the Ethics Committee of the coordinating centre (A.O.U. Città della Salute e della Scienza di Torino: Protocol n. 35258; 28/03/2022) and locally by each participating centre.

All patients provided written informed consent. Immigrants born abroad had legal residency status, granting them free access to medical care. Unauthorised asylum seekers and refugees were not considered, as they were not routinely managed in the participating centres. The participating centres collected demographic, clinical and selected virologic data as per standard clinical practice. Liver cirrhosis was diagnosed by liver stiffness measurement (LSM) (FibroScan, Echosens, France), by diagnostic clinical, imaging and biochemical features, or with a previous liver biopsy as described in the Supporting Information S1. In the absence of per‐protocol liver biopsies, inflammatory liver disease was deduced from alanine aminotransferase (ALT) values > 40 U/L with the arbitrary inference that ALT values ≤ 40 U/L stood for lack of clinical disease; however, since normal ALT may also be compatible with mild liver damage, thus the wording clinically inactive disease as used in this study does not entirely rule out underlying inflammation.

Virologic analyses performed at the local centres included the determination of quantitative HBsAg, hepatitis B e antigen (HBeAg), anti‐HDV, quantitative HBV‐DNA, antibodies to hepatitis C virus (HCV) and human immunodeficiency virus (HIV).

Serum samples collected from all patients were sent to Turin for the determination of quantitative HDV‐RNA, HDV genotype and subgenotype, HBV genotype and hepatitis B core‐related antigen (HBcrAg).

### 2.2 Virological Analysis in Turin

HDV‐RNA was quantified by RoboGene HDV‐RNA Quantification kit 2.0 (Robogene GmbH, Leipzig, Germany) using the CFX96 Real‐Time System BIO‐RAD (BIO‐RAD Laboratories, Hercules, CA, USA), with a lower limit of detection of 20 IU/mL. Viral nucleic acids were purified from 400 μL of serum samples using the EZ1 DSP virus kit on the EZ1 Advanced XL (QIAGEN GmbH, Hilden, Germany) and eluted in 60 μL of elution buffer as previously described [7].

HDV genotypes were determined by Sanger‐based sequencing (BMR‐GENOMICS, Padova, Italy) of a partial region of the viral genome (R1) spanning nucleotides 908–1265 [8]. The sequences obtained were compared to reference sequences of the eight HDV genotypes retrieved from GenBank, using the Basic Local Alignment Search Tool (BLAST). HDV subgenotypes were identified through phylogenetic analysis by MEGA11 software (https://www.megasoftware.net/), and reference sequences for subgenotyping were retrieved from Karimzadeh and colleagues [9]. Sequences were aligned using ClustaW. Pairwise genetic distance was calculated to estimate inter‐subgenotype differences, and phylogenetic trees were generated by the Neighbour‐Joining method. The reliability of interior branch lengths was estimated by a bootstrap approach (100 replicates).

HBV genotypes were determined using a reverse hybridisation line probe assay (INNO‐LiPA HBV Genotyping, Fujirebio Europe NV, Gent, Belgium), designed to identify HBV genotypes A to H by detecting genotype‐specific sequences in the HBV polymerase gene domains B and C [10].

HBcrAg was measured by chemiluminescent enzyme immunoassay (Lumipulse G HBcrAg) on a LUMIPULSE G600 II analyser (Fujirebio, Tokyo, Japan). HBcrAg values were reported as Log U/mL (measurement range: 2.0–7.0 Log U/mL) [11].

### 2.3 Statistical Analysis

Continuous variables were reported as median and interquartile range (IQR), whereas categorical parameters were reported as number (*n*) and percentages (%). The Mann–Whitney test was used to compare quantitative data between groups, whereas the chi‐squared (*χ* ^2^) test was used to compare qualitative data. The correlation between continuous variables was assessed by the Spearman correlation test (*r*_s_) with 95% confidence intervals (CI).

The statistical analysis was performed using MedCalc software v.18.9.1 (MedCalc Software Ltd. Ostend, Belgium), and *p*‐values < 0.05 were considered statistically significant.

## 3 Results

### 3.1 Patients' Characteristics

We enrolled 515 patients, comprising 317 (61.6%) native Italians (hereafter referred to as Italians) and 198 immigrants born abroad (hereafter referred to as migrants).

**FIGURE 1:**
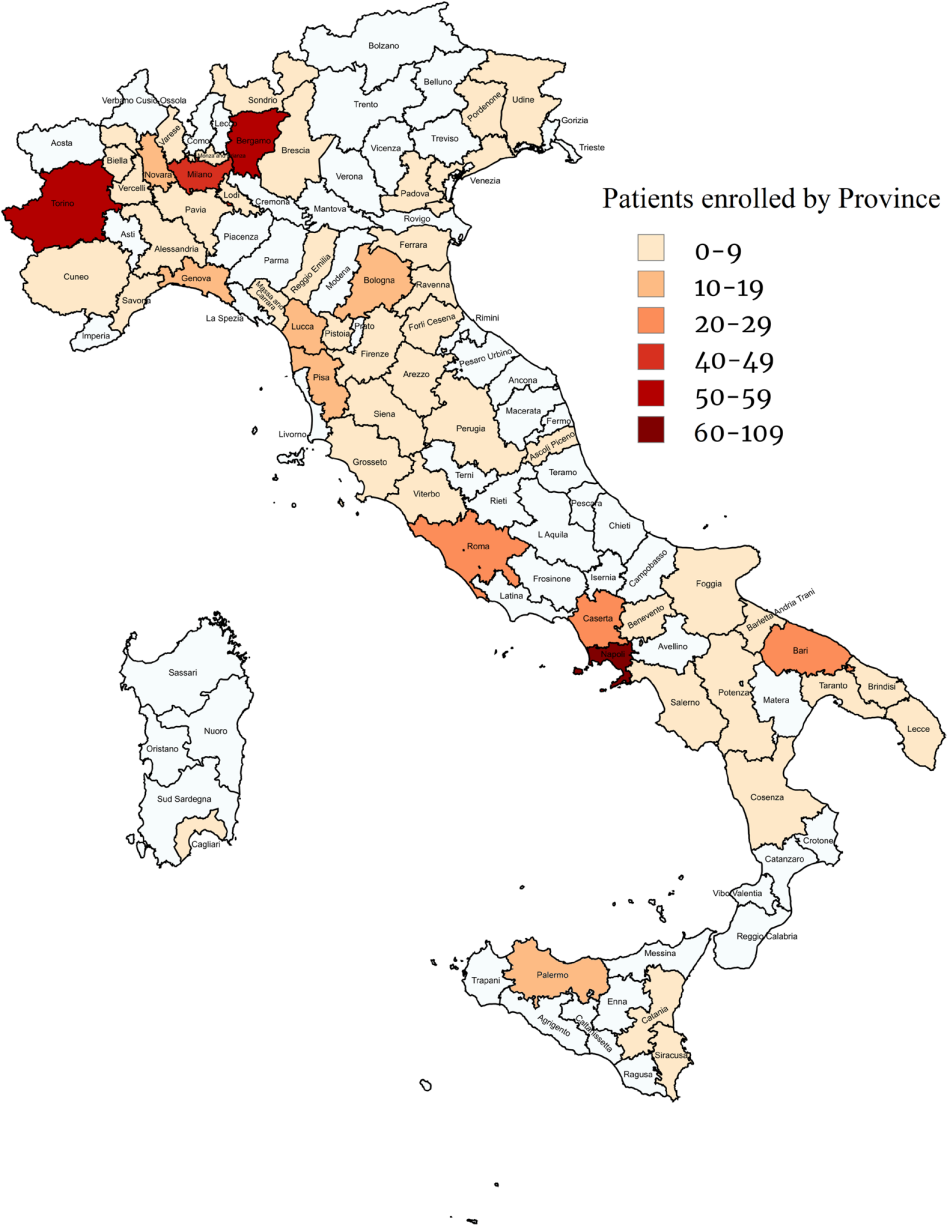
Patients' distribution according to Italian province of residence.

The overall features of the patients are shown in Table 1. Their median age was 57 (47–63) years; the majority were male. The distribution of patients according to the province of residence is shown in Figure 1; the prevalence of migrants was higher in Central/North Italy than in South Italy/Islands (177 of 305 [58.0%] vs. 21 of 210 [10.0%]; *p* < 0.001) (Figure S1). One hundred seventy (85.9%) of the migrants were from Eastern Europe (Romania: 71, 41.8%; Moldova: 59, 34.7%; Albania: 25, 14.7%; Ukraine: 11, 6.5%; Georgia: 2, 1.2%; Russia: 2, 1.2%), 19 (9.6%) from Africa (North Africa: 5, 26.3%; Sub‐Saharan Africa: 14, 73.7%), 6 (3.0%) from Asia, 2 (1.0%) from South America and 1 (0.5%) from West Europe. Three hundred twenty‐three (62.7%) patients had liver cirrhosis.

**TABLE 1:** Comparison of the demographic and clinical features of 317 Italians and 198 migrants HBsAg carriers with anti‐HDV.

Variables	Overall *n* = 515	Italians *n* = 317 (61.6%)	Migrants *n* = 198 (38.4%)	*p*
Age (years), median (IQR) [515]	57 (47–63)	60 (55–66)	46 (39–54)	< 0.001
Sex [515]				
Males, *n* (%)	317 (61.6%)	216 (68.1%)	98 (49.5%)	< 0.001
Females, *n* (%)	198 (38.4%)	101 (31.9%)	100 (50.5%)	
BMI (kg/m^2^), median (IQR) [461]	24.6 (22.6‐27.7)	24.6 (22.7‐27.5)	24.5 (22.5‐27.8)	0.711
Risk factors [515]				
Risky alcohol intake, *n* (%)*	67 (13.0%)	43 (13.6%)	24 (12.1%)	0.636
IVDU, *n* (%)	65 (12.6%)	61 (19.2%)	4 (2.0%)	< 0.001
Blood transfusion, *n* (%)	34 (6.6%)	22 (6.9%)	12 (6.1%)	0.696
HBsAg+ household members, *n* (%)	86 (16.7%)	54 (17.0%)	32 (16.2%)	0.796
Anti‐HDV+ household members, *n* (%)	47 (9.1%)	29 (9.1%)	18 (9.1%)	0.983
Anti‐HCV‐positive, *n* (%) [492]	75 (15.2%)	66 (21.6%)	9 (4.8%)	< 0.001
Anti‐HIV‐positive, *n* (%) [436]	35 (8.0%)	32 (11.6%)	3 (1.9%)	< 0.001
ALT (U/L), median (IQR) [515]	46 (28–80)	43 (26–85)	49 (29–76)	0.661
≤ 40 U/L, *n* (%)	234 (45.4%)	152 (57.9%)	82 (41.4%)	0.148
> 40 U/L *n* (%)	281 (54.6%)	165 (52.1%)	116 (58.6%)	
Platelet count (×10^9^/L), median (IQR) [512]	139 (88–197)	131 (86–184)	160 (92–208)	0.010
Albumin (g/dL), median (IQR) [505]	4.1 (3.7–4.4)	4.0 (3.7–4.3)	4.2 (3.9–4.5)	< 0.001
HDV‐RNA (Log IU/mL), median (IQR) [515]	4.39 (1.30–5.82)	4.42 (1.30–5.73)	4.21 (1.45–5.91)	0.319
Positive, *n* (%)	432 (83.9%)	261 (82.3%)	171 (86.4%)	0.227
Negative, *n* (%)	83 (16.1%)	56 (17.7%)	247 (13.6%)	
HBcrAg (Log U/mL), median (IQR) [515]	3.1 (2.3–4.3)	3.0 (2.3–3.9)	3.4 (2.5–4.6)	< 0.001
LSM by VCTE (kPa), median (IQR) [402]	11.6 (7.3–18.5)	13.2 (8.3–19.9)	9.3 (6.3–14.3)	< 0.001
Liver cirrhosis, *n* (%) [515]**	323 (62.7%)	223 (70.3%)	100 (50.5%)	< 0.001
Previous IFN treatment, *n* (%) [506]	159 (31.4%)	88 (28.6%)	71 (35.9%)	0.085
Ongoing NUC treatment, *n* (%) [515]	382 (74.2%)	247 (77.9%)	135 (68.2%)	0.014
Ongoing Bulevirtide treatment, *n* (%) [515]	31 (6.0%)	21 (6.6%)	10 (5.1%)	0.465

*Note:* Numbers in brackets indicate patients with available data. *p* values were calculated by the Mann–Whitney test for continuous variables and by *χ* ^2^ test for categorical data. Overall, 129 patients underwent liver biopsy. Liver cirrhosis was diagnosed by liver biopsy in 88 (27.2%) patients, by LSM in 125 (38.7%) patients, and by a combination of clinical, imaging and biochemical features in 110 (34.1%) patients.

Abbreviations: ALT, alanine aminotransferase; anti‐HCV, antibody to hepatitis C virus; anti‐HDV, antibodies to hepatitis D virus; anti‐HIV, antibodies to human immunodeficiency virus; BMI, body mass index; HBcrAg, hepatitis B core‐related antigen; HBsAg, hepatitis B surface antigen; HCC, hepatocellular carcinoma; HDV, hepatitis D virus; IFN, interferon; IQR, interquartile range; IVDU, intravenous drug use; LSM, liver stiffness measurement; *n*, number; NUC, nucleos(t)ide analogues against HBV; VCTE, vibration controlled transient elastography.

* > 30 g/day for males and > 20 g/day for females.

** Including 47 patients with HCC among native Italians and 1 patient with HCC among migrants.

The 317 native Italians were older than the 198 migrants (median age 60, 55–65 vs. 46, 39–54 years; *p* < 0.001), were more commonly male (male‐to‐female ratio: 2.14 vs. 0.98; *p* < 0.001) and exhibited a higher prevalence of liver cirrhosis (223 [70.3%] vs. 100 [50.5%]; *p* < 0.001) and hepatocellular carcinoma (47 [14.8%] vs. 1 [0.5%]; *p* < 0.001) (Table 1). Italians more often displayed anti‐HCV and anti‐HIV antibodies and reported more frequently a past habit of intravenous drug use (Italians: *n* = 61 [19.2%] vs. migrants: *n* = 4 [2.0%]; *p* < 0.001). No difference was observed between the two groups in previous major surgery, blood transfusions, body mass index (BMI), alcohol intake and household cohabitation with HBsAg/anti‐HDV‐positive members.

Four hundred thirty‐two (83.9%) patients were HDV‐RNA positive. Median serum HDV‐RNA was 4.39 (1.30–5.82) Log IU/mL. HDV‐RNA correlated with ALT (*r*_s_ = 0.575, 0.514–0.605; *p* < 0.001), and HBcrAg (*r*_s_ = 0.521, 0.455–0.581; *p* < 0.001); in turn, HBcrAg correlated with ALT (*r*_s_ = 0.357, 0.280–0.430; *p* < 0.001). No significant differences in correlation patterns were observed between Italians and migrants (Figure S2).

### 3.2 Comparison of the Clinical and Virologic Features of the Patients According to Liver Cirrhosis and Birthplace

Italians and migrants were stratified by the presence of liver cirrhosis and by birthplace (Table S1). Reductions in platelet counts and serum albumin were observed only in patients with cirrhosis; Italian cirrhotics had lower albumin values than migrant cirrhotics (4.0, 3.6–4.2 g/dL vs. 4.1, 3.8–4.4 g/dL; *p* = 0.012).

Independent of cirrhosis, the rate of HBeAg positivity and the median levels of HBsAg and HBcrAg were lower in Italians than migrants (respectively, 13 [4.3%] vs. 22 [11.6%], *p* = 0.002; 3.08, 1.93–3.73 Log IU/mL vs. 3.76, 2.92–4.10 Log IU/mL, *p* < 0.001; 3.0, 2.3–3.9 Log U/mL vs. 3.4, 2.5–4.6 Log U/mL, *p* = 0.001). Regardless of birthplace, patients with liver cirrhosis were more frequently on long‐term treatment with nucleos(t)ide analogues (NUCs) (276 [85.4%] vs. 106 [55.2%]; *p* < 0.001) compared to non‐cirrhotics. Among untreated patients, no significant difference in HBV‐DNA levels was observed between those with liver cirrhosis and those without (1.60, 1.02–2.34 Log IU/mL vs. 1.30, 1.06–2.05 Log IU/mL, *p* = 0.553).

At inclusion, 31 patients (6.0%), 26 with cirrhosis and 5 without cirrhosis, had just started treatment with Bulevirtide 2 mg daily via subcutaneous injection; all were HDV‐RNA positive at the time of testing. A variable proportion of patients had been previously treated with interferon (IFN), with the highest percentage in cirrhotic migrants (39.0%).

**FIGURE 2:**
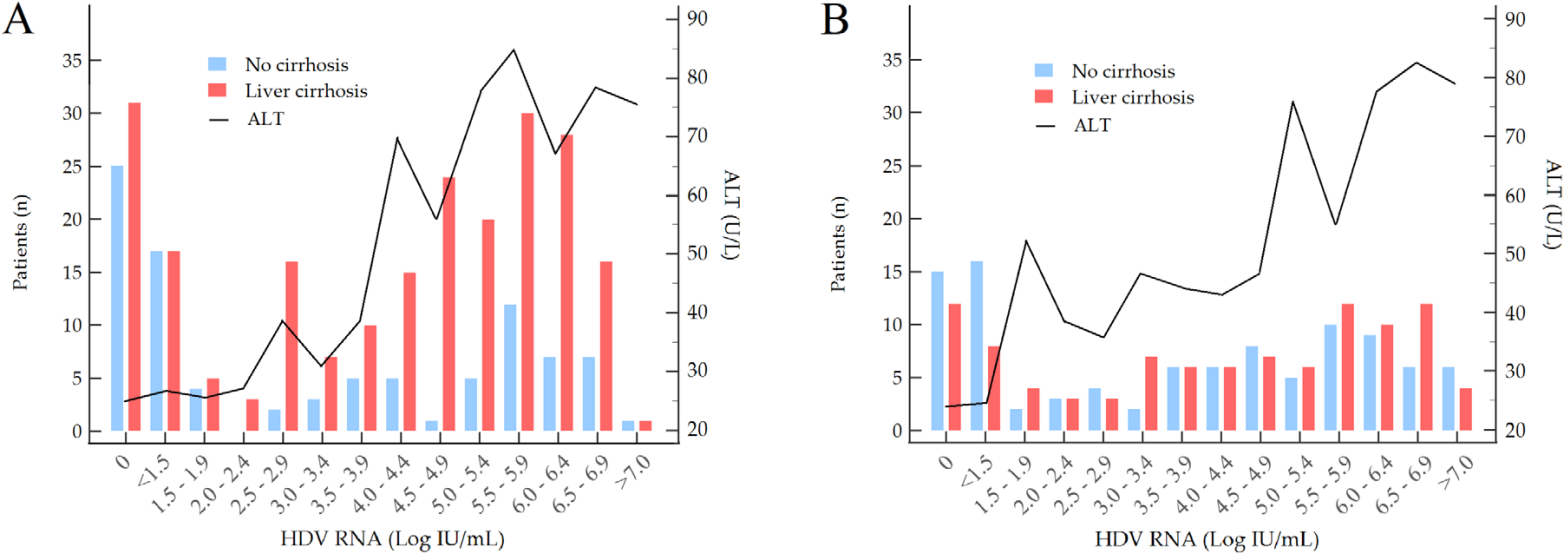
HDV‐RNA and ALT levels among Italians (A) and migrants (B) by cirrhosis status. Solid black lines indicate median ALT values across HDV‐RNA level classes. A distinct proportion of patients in both groups were either non‐viremic or had borderline viremia (< 1.5 Log IU/mL) with normal ALT. A minority exhibited marginal viremia (between 1.5 and 2.5 Log IU/mL), accompanied by normal ALT in Italians or mildly elevated ALT in migrants. Among patients with HDV‐RNA > 3 Log IU/mL, viremia increased in parallel with ALT levels. ALT, alanine aminotransferase; *n*, number.

The pattern of viremia and ALT in Italians and migrants is shown in Figure 2. Regardless of birthplace, patients with cirrhosis had higher HDV‐RNA than those without cirrhosis (4.68, 2.32–5.86 Log IU/mL vs. 3.61, 1.30–5.71 Log IU/mL; *p* = 0.006). Among Italians, 31 (13.9%) of those with cirrhosis and 25 (26.6%) of those without cirrhosis were non‐viremic with normal ALT in most. Seventeen (7.6%) of the former and 17 (18.1%) of the latter had borderline viremia (< 1.50 Log IU/mL) with normal or slightly elevated ALT. Among migrants, 12 (12.0%) of those with cirrhosis and 15 (15.3%) of those without cirrhosis were non‐viremic; 8 (8.0%) of the former and 16 (16.3%) of the latter had borderline viremia with normal or slightly elevated ALT. A minority in both groups (6 Italians and 9 migrants) had marginal viremia (< 3.00 Log IU/mL); of them, the Italians had normal ALT, whereas migrants had normal or slightly elevated ALT levels.

### 3.3 Clinical and Virologic Phenotypes

**FIGURE 3:**
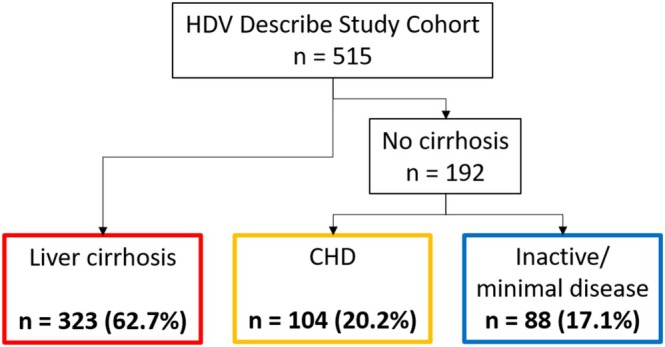
Clinical phenotypes of patients according to HDV‐RNA patterns and the presence of liver cirrhosis. Patients with inactive/minimal disease were defined by the absence of liver cirrhosis and HDV‐RNA ≤ 3.00 Log IU/mL. Abbreviations: CHD, chronic hepatitis D; HDV, hepatitis D virus; *n*, number.

Patients were stratified into three clinical and virological phenotypes on the basis of HDV‐RNA levels (above or below 3.00 Log IU/mL) and cirrhosis status: patients with liver cirrhosis (*n* = 323, 62.7%), and patients without cirrhosis, further classified into those exhibiting a clinical profile characteristic of chronic hepatitis D (CHD) (*n* = 104, 20.2%) and those presenting features consistent with inactive or minimally active disease (*n* = 88, 17.1%) (Figure 3 and Table 2).

**TABLE 2:** Demographic and clinical features of the 515 patients according to clinical phenotypes.

Variables	Liver cirrhosis *n* = 323 (62.7%)	CHD *n* = 104 (20.2%)	Inactive/minimal disease *n* = 88 (17.1%)	*p* *
Age (years), median (IQR) [515]	58 (49–64)	52 (43–61)	53 (45–61)	0.718
Sex [515]				
Males, *n* (%)	203 (62.8%)	62 (59.6%)	49 (55.7%)	0.583
Females, *n* (%)	120 (27.2%)	42 (40.4%)	39 (44.3%)	
Native Italians, *n* (%) [515]	223 (69.0%)	46 (44.2%)	48 (54.5%)	0.155
BMI (kg/m^2^), median (IQR) [461]	24.6 (22.9–28.0)	24.1 (22.0–27.0)	24.9 (22.3–28.5)	0.255
Risky alcohol intake, *n* (%)**	45 (13.9%)	14 (13.5%)	8 (9.1%)	0.345
HDV‐RNA [515]				
Negative, *n* (%)	43 (13.3%)	0	40 (45.5%)	< 0.001
≤ 3.00 Log IU/mL, *n* (%)	59 (18.3%)	0	48 (54.5%)	
> 3.00 Log IU/mL, *n* (%)	221 (68.4%)	104 (100%)	0	
ALT (U/L), median (IQR) [515]	50 (29–87)	58 (39–101)	26 (19–38)	< 0.001
HBsAg (Log IU/mL), median (IQR) [478]	3.50 (2.15–4.00)	3.77 (3.21–4.18)	2.23 (1.20–3.37)	< 0.001
HBcrAg (Log U/mL), median (IQR) [515]	3.2 (2.5–4.2)	4.1 (3.0–4.8)	2.5 (2.0–2.9)	< 0.001
LSM by VCTE (kPa), median (IQR) [402]	16.0 (12.0–25.0)	7.9 (6.2–10.6)	5.7 (4.8–7.5)	< 0.001
Previous IFN treatment, *n* (%) [506]	102 (31.6%)	35 (33.7%)	22 (25.0%)	0.192
Ongoing NUC treatment, *n* (%) [515]	276 (85.4%)	69 (66.3%)	37 (42.0%)	< 0.001
Ongoing Bulevirtide treatment, *n* (%) [515]	29 (8.0%)	5 (4.8%)	0	0.038

*Note:* Numbers in brackets indicate patients with available data. *p* values were calculated by the Mann–Whitney test for continuous variables and by *χ* ^2^ test for categorical data.

Abbreviations: ALT, alanine‐aminotransferase; BMI, body mass index; CHD, chronic hepatitis D; HBcrAg, hepatitis B core‐related antigen; HBsAg, hepatitis B surface antigen; HBV, hepatitis B virus; HDV, hepatitis D virus; IFN, interferon; IQR, interquartile range; LSM, liver stiffness measurement; *n*, number; NUC, nucleos(t)ide analogues against HBV; VCTE, vibration controlled transient elastography.

* CHD versus inactive/minimal disease.

** > 30 g/day for males and > 20 g/day for females.

Compared to patients with CHD, those with inactive/minimal disease exhibited normal ALT values (26, 19–38 U/L, *p* < 0.001 vs. 58, 39–101 U/L, *p* < 0.001), lower HBsAg (2.23, 1.20–3.37 Log IU/mL vs. 3.77, 3.21–4.18 Log IU/mL, *p* < 0.001), lower HBcrAg (2.5, 2.0–2.9 Log U/mL vs. 4.1, 3.0–4.8 Log U/mL, *p* < 0.001) and lower LSM (5.7, 4.8–7.5 kPa vs. 7.9, 6.2–10.6 kPa, *p* < 0.001).

Notably, no significant differences were observed in age, sex, birthplace or potential cofactors associated with liver damage. Furthermore, the proportion of patients who had previously received IFN therapy was comparable between patients with CHD and those with inactive/minimal disease. None of the patients in the latter group were on Bulevirtide at study inclusion.

### 3.4 Molecular HDV and HBV Epidemiology

**FIGURE 4:**
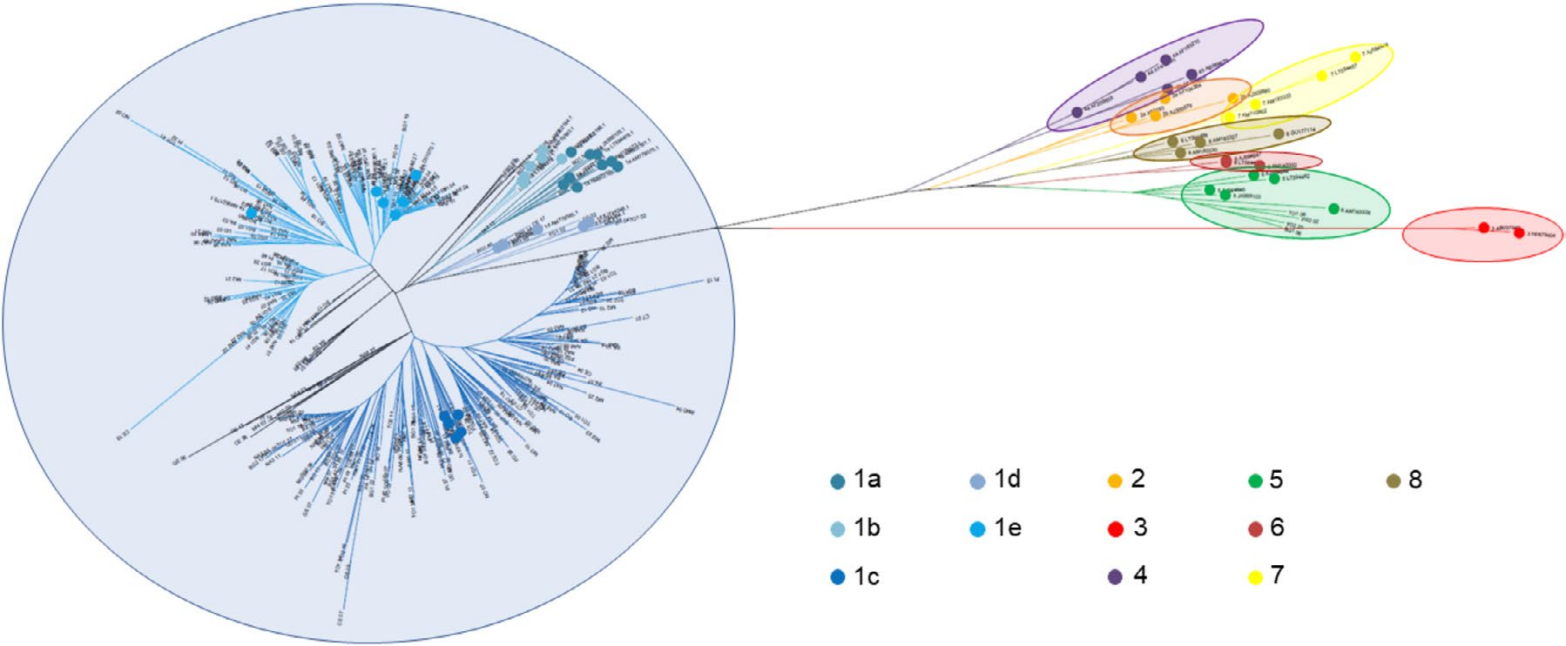
Phylogenetic analysis of HDV sequences. Phylogenetic tree of 446 HDV nucleotide sequences, including 55 reference sequences (indicated by coloured dots). HDV‐1 subgenotypes are highlighted in shades of blue, whereas sequences shown in black (*n* = 24) represent borderline cases for which subgenotype assignment was not possible. Taxa highlighted in orange correspond to HDV‐2, red to HDV‐3, purple to HDV‐4, green to HDV‐5, brown to HDV‐6, yellow to HDV‐7 and sage to HDV‐8. The evolutionary history was inferred using the Neighbour‐Joining method, and all evolutionary analyses were performed in MEGA11.

HDV sequences were obtained from 411 of 432 HDV‐RNA‐positive serum samples. Phylogenetic analysis identified HDV genotype‐1 in 407 (99.0%) patients, whereas 4 (1.0%) patients from Sub‐Saharan Africa regions were infected with HDV genotype‐5 (Figure 4).

Among patients carrying HDV genotype‐1, the HDV subgenotype was determined in 363: 1a in 4 (1.1%), 1b in 1 (0.3%), 1c in 200 (55.1%), 1d in 10 (2.8%) and 1e in 148 (40.8%). Among Italians (*n* = 216), the prevalent subgenotype was HDV‐1e (126/216; 58.3%), whereas migrants (*n* = 147) were mostly infected with HDV‐1c (119/147; 81.0%) (*p* < 0.001).

HBV genotype was determined in 86 HBV‐DNA‐positive patients. The prevalence of the different HBV genotypes did not vary between Italians and migrants (*p* = 0.309). Genotype D was the most prevalent (*n* = 62; 73.1%). Genotype A was detected in 8 patients (9.3%), C in 1 (1.2%), E in 3 (3.5%), and F in 1 (1.2%). Eleven (12.8%) patients had mixed HBV genotypes (D + C: *n* = 1; D + E: *n* = 1; D + G: *n* = 9).

## 4 Discussion

The demographics of Italians and migrants with HDV infection were previously described [12, 13], but no comprehensive analysis has thoroughly addressed their clinical characteristics. This study provides a cross‐sectional overview of the contemporary medical impact of HDV infections in Italy, on the basis of the virologic and clinical profiles of 515 HBsAg carriers with antibodies to HDV recruited from 32 medical centres between August 2022 and July 2024; the cohort included 317 individuals born in Italy and 198 migrants born abroad. Participants were newly diagnosed or in programmed follow‐up. Migrants were legally residing in Italy with access to free medical services. Unauthorised asylum seekers and refugees were not considered, meaning the migrant group represents a selected population, distinct from undocumented migrants who may have more severe clinical features; however, they reflect the migrant population with HDV infection that most impacts national healthcare systems. The majority of the patients were cirrhotic, had active disease and were HD viremic. HDV‐RNA levels correlated with active CHD and cirrhosis; 16.1% of the subjects were not viremic.

The comparison of the two populations confirmed the significantly older age and higher cirrhosis rate among Italians [12, 13]. Migrants were more prevalent in Northern and Central Italy than in Southern and Insular Italy, consistent with national immigration trends, where 58.6% of foreign citizens reside in Northern Italy, 24.5% in Central Italy and 16.9% in Southern and Insular Italy [14]. Unlike most CHD studies, where males predominate, in our study, females tend to be more prevalent than males among migrants. A possible explanation is that women typically undergo anti‐HDV testing during early pregnancy as part of mandatory HBV screening follow‐up in Italy [6, 15]. Additional risk factors for liver disease were similarly distributed between the two groups. There was no difference in HDV genotype, which was predominantly type 1 in both groups, with subgenotype HDV‐1c more common among migrants and HDV‐1e more common among Italians. HCV and HIV serology were significantly more positive in Italians, reflecting the risk of parenteral exposure to HDV through injecting drug use, a major transmission route for the virus among Italian youth in the past; many patients (74.2%) were on long‐term antiviral treatment with NUCs, and HBV‐DNA was undetectable or below the quantification limit (< 20 IU/mL) in the majority of patients. In contrast, HBcrAg was often detectable, and higher levels correlated with more advanced liver disease, as well as elevated ALT and HDV‐RNA levels. Given that the pathogenicity of HDV may depend on the transcriptional activity of the underlying HBV infection, regardless of HBV replication suppression [16, 17], HBcrAg may serve as a useful prognostic marker of advancing CHD in combination with HDV‐RNA measurement.

Age and cirrhosis differences between Italians and migrants reflect distinct epidemiological backgrounds. Younger migrants represent an ongoing infection in their countries of origin, whereas native Italians reflect the aftermath of an infection that was endemic in Italy decades ago.

Cirrhosis was observed in 70.3% of the 317 Italians, showing a dual pattern of active and inactive disease. This is consistent with the natural history of HDV cirrhosis, which can evolve from an inflammatory/viremic to a burnt‐out/non‐virulent condition; HDV‐RNA was undetectable in 13.9% of patients with cirrhosis. Previous studies have reported spontaneous clearance and reduction of HDV viremia over time. For instance, serum HDV‐RNA declined by ≥ 2 Log IU/mL in 25% of 56 Spanish cirrhotics after a mean follow‐up of 5.6 years (with 20% becoming undetectable) [18]; in Italy, HDV‐RNA was no longer detectable in 25% of 112 cirrhotics after 8 years [5], and in 85% of 21 cirrhotics after 15 years of monitoring [19].

Though less prevalent than in Italians, cirrhosis was also common among migrants, affecting 50.5% of cases. Almost 40% of these patients had been treated with IFN; although treatment did not prevent the development of cirrhosis, it may have accelerated subsequent normalisation of ALT and clearance of HDV‐RNA, which was undetectable in 12.0% of the cases.

We identified 192 patients without cirrhosis; irrespective of birthplace, half of them had elevated ALT levels and exhibited a typical CHD profile with high viremia (> 3 Log IU/mL). The other half had normal ALT levels but different virological characteristics: HDV viremia was undetectable in 45.5% of patients, indicating a past HDV infection that had resolved, whereas the other 54.5% had low‐level viremia, typically HDV‐RNA ≤ 3 log IU/mL. Notably, the discovery of spontaneously resolved infections in a distinct proportion of patients contrasts with the rare occurrence of self‐limited infections reported in previous clinical studies of HDV. This is likely attributable to the growing use of reflex testing for anti‐HDV in all HBsAg‐positive individuals [20, 21], which broadens the epidemiological context of HDV infections, leading to the identification of asymptomatic HDV cases that were previously overlooked.

The data of this study modify our understanding of CHD as an invariably severe and progressive disease. First, in Italians exposed to the HDV two to three decades before, non‐cirrhotic CHD developed as a slowly progressing liver disease of long‐standing infection, indicating that insidious forms of CHD may play a more significant role in the medical HDV landscape than previously perceived. Indolent or slowly progressive forms of HDV disease were formerly unrecognised because clinical attention focused primarily on aggressive CHD, which led to the conclusion that HDV infection was predominantly severe [22, 23]. However, with extended monitoring, more benign forms of long‐lasting CHD are now being identified, owing to their selective accumulation over time as a result of a long natural history and survival advantage. More intriguing, 48 patients had low‐level viremia, typically equal to or lower than 3 log IU/mL, but exhibited normal ALT values. The significance of minor viremia without overt liver injury cannot be interpreted from this cross‐sectional study, which only offers a snapshot of the natural history of HDV infection. However, in contrast to the belief that the expression of HDV‐RNA results in important liver damage [23], this finding raises the question whether spontaneous low‐grade HDV viremia may also be compatible with indolent clinical conditions; in a recent study, untreated HDV patients with low levels of HDV‐RNA also had fewer clinical outcomes [24].

Second, the high rate of cirrhosis in migrants is somewhat surprising, as one would assume that the younger age of the migrants should have driven primarily fresh and florid forms of CHD. This suggests that a proportion of CHD rapidly progressed to cirrhosis but subsequently followed a stable clinical course, in line with similar findings in other series of migrants in Europe. In France and Sweden [25, 26], cirrhosis was found at referral in 23.4% and 28.1% of 1112 and 337 patients, largely composed of migrants, with a mean age of 36.5 and 38 years, respectively; in both studies, the further clinical progression appeared to be less severe than predicted from the presumptive ominous course of HDV disease.

The relatively milder medical impact of HDV in migrants to Europe suggested that the profile of patients with CHD might have shifted to a more benign clinical course because of extended testing with reduced selection bias toward severe liver disease [27]. However, this assumption is difficult to reconcile with the significant proportion of young migrants diagnosed with cirrhosis. Rather, young, labour‐fit migrants with anti‐HDV cirrhosis who come to Europe may represent a selected population with less severe clinical disease, which does not fully reflect the medical burden of HDV in their home countries; those with severe, debilitating CHD would likely be unable to emigrate. Although observational studies emphasise the tendency of CHD to rapidly progress to cirrhosis [28, 29, 30], the cirrhotic outcome may be followed by a prolonged, stable clinical course. This variant, similar to the cirrhosis seen in migrants, was first described in Italian cirrhotics who survived symptom‐free for an average of 22 years before clinical decompensation [3]. The proportion of this variant within the broader CHD scenario and the reason for its prolonged course remain unclear. A hypothesis may be that many of these young patients were originally healthy, inactive HBsAg carriers, whose livers were better able to withstand the damage caused by HDV superinfection compared to HBsAg carriers with underlying HBV liver disease.

The example of Italy serves as a paradigm for the current approach to HDV in high‐income countries that are increasingly attracting immigrants; addressing the infection requires consideration of the differing medical characteristics between natives and newcomers.

## Author Contributions

M.R. contributed to the conceptualization and study design, data analysis, data interpretation and writing of the manuscript. G.P.C. contributed to study design, data acquisition, data analysis, data interpretation and writing of the manuscript. T.S. contributed to the study design and data analysis. G.P.C., E.D., A.O. and G.M. performed laboratory analysis. A.L., M.R.B. and G.A.N. critically revised the manuscript for important intellectual content. A.C., A.L., M.V., S.F., P.C., B.C., D.A., G.G.D.C., C.M., G.R., V.M., M.M., A.S., L.M., G.C., V.C., F.M., S.C., G.V., E.B., G.D., M.F., E.P., C.C., R.R., M.P., A.I., T.S., V.D.M., A.M., N.C., M.R., A.M.C., G.A.N., P.T., F.R.P., L.S.B., A.F., G.B., M.B., M.P., A.C., M.D., L.A.S., R.C., E.M.C., M.A. and P.L. were responsible for patient recruitment, patient care, and data collection. G.P.C., E.D., A.O. and M.R. had access to and verified all the underlying data reported in the manuscript. All authors approved the final version of the manuscript and accept responsibility to submit for publication.

## Conflicts of Interest

G.P.C. reports grants from Fujirebio Diagnostics AB; A.L. reports consulting and/or speaker fees from Gilead Sciences. M.V. reports consulting and/or speaking fees from Gilead Sciences, and Ipsen. S.F. reports consulting and/or speaking fees from Gilead Sciences, Roche and Alfasigma. All other authors report no potential conflicts.

## Supporting information


**Data S1:** liv70242‐sup‐0001‐supinfo.docx.

## Data Availability

The data that support the findings of this study are available from the corresponding author upon reasonable request.
