# Supplementary material for: The Medical Impact of Hepatitis D Virus Infection in Natives and Immigrants: The Italian Paradigm
Source: Liver Int. 2025 Jul 29;45(9):e70242. doi: 10.1111/liv.70242 (PMC12306849; doi:10.1111/liv.70242)
Supplement: Supplementary file 1 — Data S1: liv70242‐sup‐0001‐supinfo.docx. [file LIV-45-0-s001.docx]

**Supplementary Materials**

**Table of content**

Supplementary Materials and Methods………………………………………………………………………………….....2

Supplementary Table 1………………………………………………………………………………………………….…3

Supplementary Figure 1…………………………………………………………………………………………………....5

Supplementary Figure 2………………………………………………………………………………………………..…..6

**SUPPLEMENTARY MATERIALS AND METHODS**

**Diagnostic criteria for liver cirrhosis**

The diagnosis of liver cirrhosis was established based on the following criteria:

1. Evidence of cirrhosis by liver biopsy (Ishak ≥ 5 or Metavir = 4),

or

1. Liver stiffness measurement by vibration-controlled transient elastography (FibroScan®, Echosens™, France) ≥ 12·5 kPa,^1^

and/or

1. Radiological features indicative of liver cirrhosis, including hepatic nodularity and splenomegaly observed on ultrasound or computed tomography,

and/or

1. Clinical, endoscopic, and laboratory findings indicative of portal hypertension including:

a. Ascites

b. Hepatic encephalopathy

c. Oesophageal or gastric varices at upper endoscopy

d. Thrombocytopenia (platelet count <150 x 10^9^ /L).^2^

**References**

1. European Association for the Study of the Liver. EASL Clinical Practice Guidelines on non-invasive tests for evaluation of liver disease severity and prognosis - 2021 update. J Hepatol 2021;75:659-89.
2. De Franchis R, Bosch J, Garcia-Tsao G, *et al*. Baveno VII - Renewing consensus in portal hypertension. J Hepatol 2022;76:959-74.

**SUPPLEMENTARY TABLE 1** Comparison of clinical and virologic features of the 515 HBsAg positive patients with anti-HDV divided by presence or absence of liver cirrhosis and birthplace.

|  | **Italians** | | **Migrants** | |  |  |  |  |
| --- | --- | --- | --- | --- | --- | --- | --- | --- |
| **Variables** | **Cirrhosis**  ***n*=223 (43.3%)** | **No cirrhosis**  ***n*=94 (18.3%)** | **Cirrhosis**  ***n*=100 (19.4%)** | **No cirrhosis**  ***n*=98 (19.0%)** | ***p*-value*** | ***p*-value**** | ***p*-value^†^** | ***p*-value^‡^** |
| Age (years), median (IQR) [515] | 61 (56–67) | 59 (52–65) | 46 (40–55) | 46 (36–52) | **.**026 | **.**135 | <**.**001 | <**.**001 |
| Sex [515]   - Males, *n* (%) - Females, *n* (%) | 153 (68**.**6%)  70 (31**.**4%) | 63(67**.**0%)  31 (33**.**0%) | 50 (50**.**0%)  50 (50**.**0%) | 48 (49**.**0%)  50 (51**.**0%) | **.**782 | **.**886 | **.**001 | **.**012 |
| LSM by VCTE (kPa), median (IQR) [402] | 16**.**6 (12**.**4-25**.0**) | 7**.**5 (5**.**3–10**.**3) | 15**.**4 (12**.0**–246) | 6**.**6 (5**.**4–8**.**0) | <**.**001 | <**.**001 | **.**620 | **.**033 |
| ALT (U/L), median (IQR) [515] | 46 (28–87) | 39 (23–80) | 57 (33–88) | 44 (25–61) | **.**187 | **.**001 | **.**078 | **.**699 |
| - ≤40 U/L, *n* (%) - >40 U/L, *n* (%) | 100 (44**.**8%)  123 (55**.**2%) | 52 (55**.**3%)  42 (44**.**7%) | 35 (34**.**0%)  66 (66**.**0%) | 48 (49**.**0%)  50 (51**.**0%) | **.**089 | **.**033 | **.**068 | **.**381 |
| Platelet count (x 10^9^/L), median (IQR) [512] | 100 (73-156) | 193 (164-240) | 106 (67-140) | 199 (169–238) | <**.**001 | <**.**001 | **.**539 | **.**794 |
| Albumin (g/dL), median (IQR) [505] | 4**.**0 (3**.**6–4**.**2) | 4**.**2 (3**.**9–4**.**4) | 4**.**1 (3**.**8–4**.**4) | 4**.**2 (4**.**0–4**.**5) | **.**001 | **.**009 | **.**012 | **.**065 |
| HBsAg (Log IU/mL), median (IQR) [478] | 3**.**26 (1**.**99–3**.**85) | 2**.**78 (1**.**62–3**.**58) | 3**.**79 (3**.**21–4**.**09) | 3**.**73 (2**.**80–4**.**16) | **.**045 | **.**918 | <**.**001 | <**.**001 |
| HBeAg-positive, *n* (%) [495] | 10 (4**.**6%) | 3 (3**.**4%) | 11 (11**.**3%) | 11 (11**.**8%) | **.**639 | **.**917 | **.**028 | **.**035 |
| HBcrAg (Log U/mL), median (IQR) [515] | 3**.**1 (2**.**3–4**.**0) | 2**.**8 (2**.**0–3**.**6) | 3**.**4 (2**.**7–4**.**4) | 3**.**4 (2**.**0–4**.**7) | **.**104 | **.**612 | **.**010 | **.**014 |
| - ≤3**.**0 Log U/mL, *n* (%) - >3**.**0 Log U/mL, *n* (%) | 107 (48**.**0%)  116 (52**.**0%) | 57 (60**.**6%)  37 (39**.**4%) | 38 (38**.**0%)  62 (62**.**0%) | 45 (45**.**9%)  53 (54**.**1%) | **.**040 | **.**260 | **.**096 | **.**042 |
| HDV-RNA (Log IU/mL), median (IQR) [515] | 4**.**73 (2**.**38–5**.**79) | 2**.**73 (0–5**.**69) | 4**.**53 (2**.**10–6**.**03) | 4**.**06 (1**.**30–5**.**80) | **.**005 | **.**200 | **.**503 | **.**076 |
| - Negative, *n* (%) - ≤3**.**00 Log IU/mL, *n* (%) - >3**.**00 Log IU/mL, *n* (%) | 31 (13**.**9%)  41 (18**.**4%)  151 (67**.**7%) | 25 (26**.**6%)  23 (24**.**5%)  46 (48**.**9%) | 12 (12**.**0%)  18 (18**.**0%)  70 (70**.**0%) | 15 (15**.**3%)  25 (25**.**5%)  58 (59**.**2%) | **.**001 | **.**169 | **.**627 | **.**062 |
| Previous IFN treatment, *n* (%) [506] | 63 (28**.**6%) | 25 (28**.**4%) | 39 (39**.**0%) | 32 (32**.**7%) | **.**968 | **.**353 | **.**066 | **.**532 |
| Ongoing NUC treatment, *n* (%) [515] | 189 (84**.**8%) | 58 (61**.**7%) | 87 (87**.**0%) | 48 (49**.**0%) | <**.**001 | <**.**001 | **.**597 | **.**077 |

Numbers in brackets indicate patients with available data. p values were calculated by Mann-Whitney test for continuous variables and by χ^2^ test for categorical data.*Native Italians: liver cirrhosis vs no cirrhosis; **Migrants: liver cirrhosis vs. no cirrhosis; ^†^Liver cirrhosis: native Italians vs. migrants; ^‡^No cirrhosis: native Italians vs. migrants.

Abbreviations: ALT, alanine-aminotransferase; anti-HDV, antibodies to hepatitis D virus; HBcrAg, hepatitis B core-related antigen; HBeAg, hepatitis B e antigen; HBsAg, hepatitis B surface antigen; HBV, hepatitis B virus; HDV, hepatitis D virus; IFN, interferon; IQR, interquartile range; LSM, liver stiffness measurement; *n*, number; NUC, nucleos(t)ide analogues against HBV; VCTE, vibration controlled transient elastography.

**SUPPLEMENTARY FIGURE 1** Distribution of Italians (A) and migrants (B) according to Italian province of residence.

**
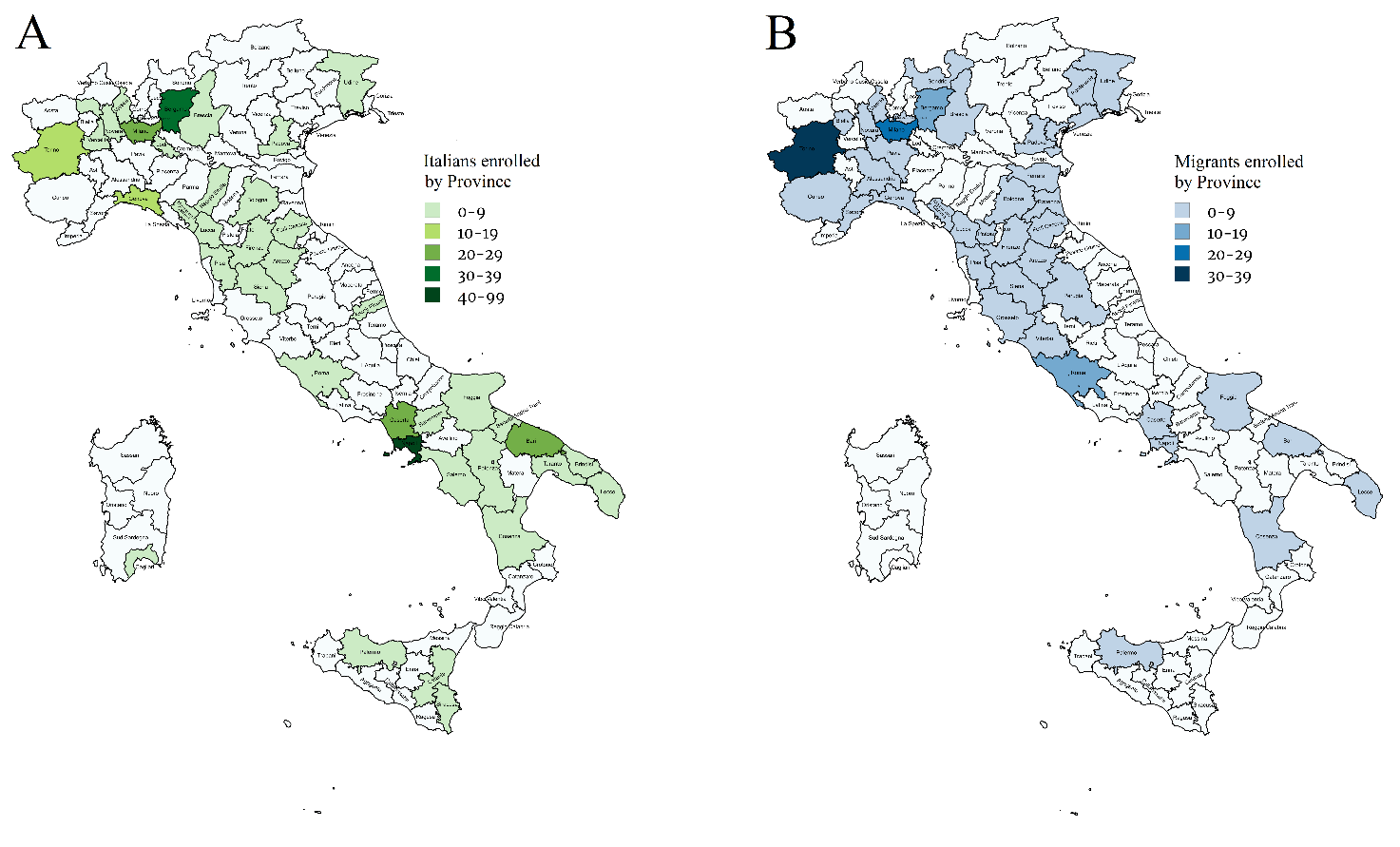
**

The ethnic composition of the migrant population was as follows: Caucasian (*n*=171), Sub-Saharan African (*n*=14), North African (*n*=5), Asian (*n*=6), and Andean Indigenous (*n*=2).

**SUPPLEMENTARY FIGURE 2** Rate of HDV-RNA positivity (red bar) (A) and correlation between HDV-RNA and ALT (B), LSM (C), HBcrAg (D), and between HBcrAg and ALT (E), and LSM (F).

**
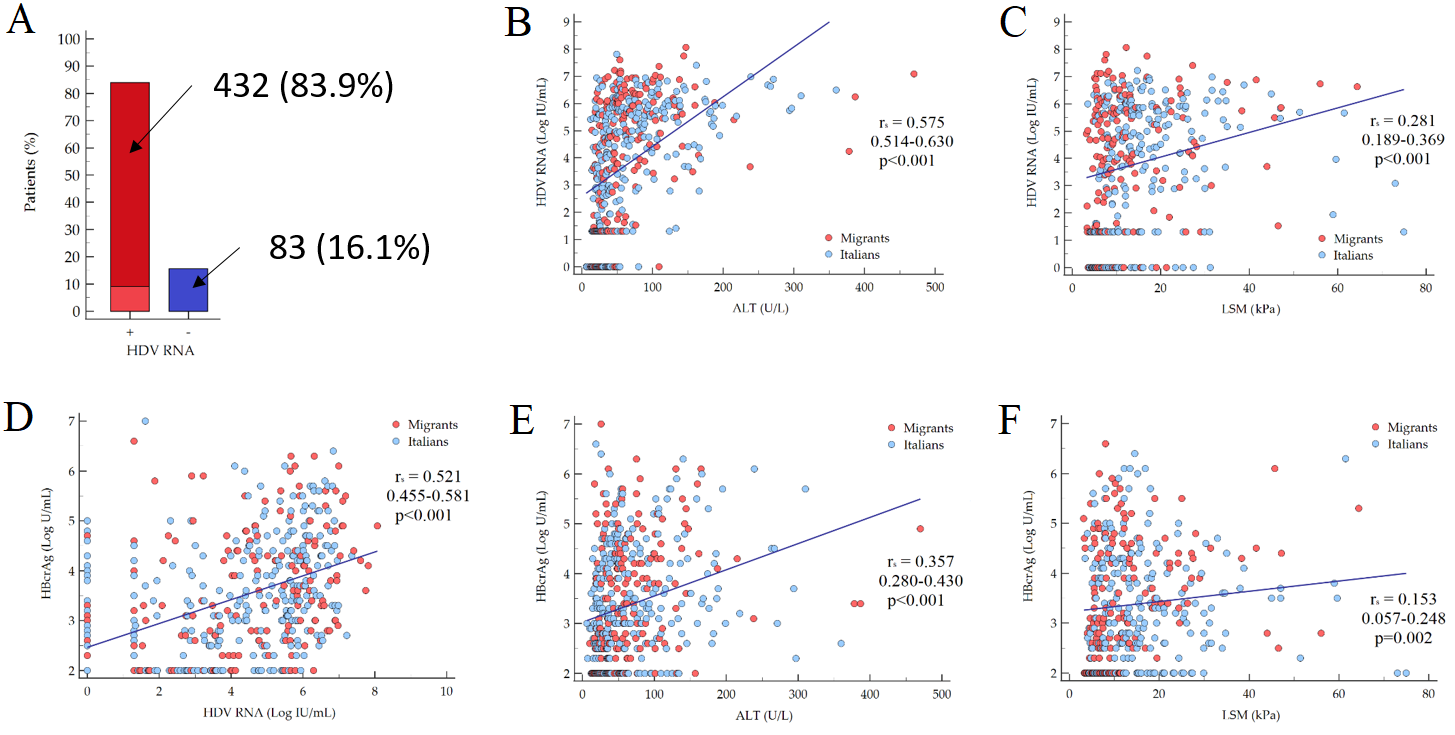
**

Italians: blue dots; migrants: red dots.

Abbreviations: ALT, alanine aminotransferase; HBcrAg, hepatitis B core-related antigen; HDV, hepatitis D virus; LSM, liver stiffness measurement.
